# Psychological Distress in Elite Sambo and Recreational Athletes

**DOI:** 10.3389/fpsyg.2022.781880

**Published:** 2022-04-29

**Authors:** Tatjana Tubić, Bogdan Živanović, Nemanja Lakićević, Nataša Zenić, Barbara Gilić, Eduardas Rudas, Sergey Eliseev, Tatjana B. Trivić, Antonino Bianco, Patrik Drid

**Affiliations:** ^1^Faculty of Sport and Physical Education, University of Novi Sad, Novi Sad, Serbia; ^2^Sport and Exercise Sciences Research Unit, University of Palermo, Palermo, Italy; ^3^Faculty of Kinesiology, University of Split, Split, Croatia; ^4^Faculty of Kinesiology, University of Zagreb, Zagreb, Croatia; ^5^Martial Arts Academy, Vilnius, Lithuania; ^6^Institute of Sport and Physical Education, Russian State University of Physical Education, Sports, Youth and Tourism, Moscow, Russia

**Keywords:** psychological distress, athletes, elite sambo, recreational, prevalence

## Abstract

**Background:**

Previous studies suggest that engagement in any type of physical activity can be protective against mental health issues, whereas elite-level athletes can endure various mental health challenges. The aim of this study was to determine variations in the prevalence of psychological distress among elite sambo athletes and their recreational counterparts.

**Methods:**

A sample consisting of 245 athletes (127 males and 118 females) was chosen. Out of the total sample, 105 were elite-level athletes while 140 were recreational athletes. Participants were accessed via the Depression Anxiety Stress Scales-21 to determine their stress in various domains at a given time.

**Results:**

Data indicated that all tested differences between elite sambo athletes and recreational athletes were statistically significant; recreational athletes had a higher score on the depression scale, anxiety and stress, and a general distress score than sambo athletes. Although there are no gender differences in psychological distress in the total sample of athletes, elite sambo athletes achieve significantly lower scores in all tested variants than recreational ones. Women who engage in recreational activities have stood out as a vulnerable subsample in psychological stress.

**Conclusion:**

Future epidemiological and interventional studies should explore optimal strategies to identify mental health needs based on specific sport activity, especially in terms of gender. There is a need to place special emphasis on psychological distress in the context of combat sports.

## Introduction

Despite the essential importance of mental health in the athletic population for achieving outstanding performance (Purcell et al., 2019), little is known about their mental health, particularly with respect to the prevalence of mental health issues. Lack of evidence in this field has commonly been justified on the stereotypical basis that athletes are just “stronger people” and that sport naturally selects individuals in terms of health, both physical and psychological (Chang et al., 2020). Several studies confirm these claims to a degree; athletes are less depressive and anxious compared with the overall population and adapt to stressful situations easier, have more self-esteem, and have better body image (Rice et al., 2016; Gouttebarge et al., 2019). However, it is being neglected that the athletic environment often includes unique stressors caused by high-pressure circumstances, constant mental efforts, demands, and limitations which may negatively impact mental health (Rice et al., 2016). In addition, professional sport is completely different from recreational sport with regard to mental health risk factors (Gouttebarge et al., 2017). To affirm the aforementioned, studies have repeatedly shown that athletes who constantly have to prove themselves to a coach and/or teammates have better career trajectories than their counterparts playing the same position, as those who are unhappy with the development of their sports career or were injured and are preparing to end their career (usually those who are the most committed and at elite level) (Gouttebarge et al., 2019). It is often forgotten that the picture of athletes' mental health is formed based on the number of those who request help (Prinz et al., 2016), but there are certainly those who neglect the problem and do not even ask for help. In addition, there is a lack of educative effort among coaches, physicians, physiotherapists, and club managers who are in permanent and in direct contact with athletes as well as those who are supposed to recognize and prevent mental health problems of their players (Prinz et al., 2016). If the stigma is added to already bad mental health situations in athletes (Bauman, 2016), which is connected to the acknowledgment that mental health issues among athletes exist and which is further extended to athletes' status within teammates, coaches, fans, and social interaction, it becomes inevitable to confess that mental health of athletes undoubtedly becomes an interplay where inter- and intrapersonal factors are perplexed. Depending on which of these factors is dominant, outcomes of mental health are better or worse, and this should be the key aspect of mental health research in athletes.

Early detection of mental health symptoms in athletes has to take into consideration the prevalence of mental health symptoms, which is based on gender and/or (specific) sport practiced. As far as gender-dependent mental health problems are concerned, epidemiological studies consistently indicate that females are more susceptible to mental health issues than males (Leach et al., 2008). Contingent on the type of mental issue, prevalence among females is two times higher for depression for anxiety and multiple times higher (up to 10 times higher) for eating disorders compared to males (Brody and Hall, 2008; Leach et al., 2008; Schaal et al., 2011). Relative inconsistency of these findings in the athletic population (Kotnik et al., 2012; Rice et al., 2016; Akesdotter et al., 2020) was found due to methodological differences in various studies (different measurement tools, specific sports samples, and other sports-related variables) and until now has not questioned the position that mental health problems in athletes and elite athletes show equal gender dependence like in general population (Schaal et al., 2011).

Specific-sport-based differences also reflect on aspects of mental health. For instance, significant differences in the level of anxiety which exist among athletes who participate in gymnastics or figure skating or those who engage in high-risk sports (Schaal et al., 2011; Lakicevic et al., 2020) can be explained by the specificity of these sports. In the first instance, success depends on the jury decision, and tension develops as the control of the situation is decreased. In the second group of sports, seeking excitement and going against danger is the essence of activity, so anxiety or fear would represent a counter indicator of involvement in that particular sport (and exceptions would just affirm that rule). Aiming toward perfection, known to be present in artistic swimming, can hardly be achieved in other sports. Regarding eating disorders, prevalence varies in different sports with exceptionally high levels of racing and fine motor skills when matched against team ball sports. For male athletes, however, participation in combat and contact sports showed the highest prevalence of eating disorders in comparison with the type of sports (Schaal et al., 2011; Lakicevic et al., 2021).

The commonality of all mental health problems, given all gender and/or sports type and/or sports level, is the presence of unpleasant emotions (Werner and Gross, 2004), with the absence of those problems being unthinkable to diagnose any problem of mental health. Therefore, unpleasant emotions or emotional conditions which threaten to overwhelm us and that affect our everyday activities and relationships with other people jointly termed psychological distress—are known to be the most reliable markers of measuring mental health status (Diener et al., 2010). When a person is constantly facing situations that cause discomfort, fear, or worry and is unable to confront those, that person can start avoiding social contact, feels tired, sad, or angry and is overpowered, which consequently leads to poor performance and also impact existing conditions negatively (Werner and Gross, 2004). If affected persons are athletes, timely intervention can have a profound impact on their career trajectories (Wylleman et al., 2015).

Predicated on the findings that the international success of juniors in combat sports represents a significant predictor of the long-term international success of seniors (Li et al., 2018), early identification of susceptibility elements of psychological distress in younger age categories enables eradication or mitigation of its effect on sports performance and benefits the overall health of an athlete. This also holds true in senior age athletes, because, if neglected, psychological distress can become an even great problem after a career (Prinz et al., 2016).

The main aim of this study was to analyze variations in the prevalence of psychological distress based on gender and sport practiced among elite sambo athletes and recreational athletes, particularly in the measurements of depression, anxiety and stress, and general distress, in overall distress with a particular emphasis on sex-based differences. Based on the previous findings on variations in the prevalence of mental health symptoms among athletes of both gender in specific sports, we hypothesize that differences in the examined aspects of mental health between elite sambo and recreational athletes exist; gender, in addition, may contribute to understanding these differences in our particular subsamples.

## Materials and Methods

### Participants

The sample included 245 athletes, of which 127 were males and 118 were females. Further, 105 were elite sambo athletes, whereas 140 participants were recreational athletes. Among elite sambo athletes (24.32 ± 6.23 years), 52 were males and 53 were females, while among recreational athletes (26.74 ± 5.30 years), 75 were males and 65 were females. Elite sambo athletes subsample were participants of the European Sambo Championship held in May 2021 in Cyprus. Sambo athletes were coming from Russia (33.3%), Uzbekistan (20.9%), Ukraine (18.1%), Lithuania (14.3%), Serbia (8.6%), and Croatia (4.8%). The average weekly exercise volume was 24.0 ± 4.2 h. Recreational athletes were coming from a fitness center in Novi Sad, Serbia, of which 25% train two to three times per week, 61.4% train four to five times per week, while 13.6% train six times per week.

To test athletes partaking in the European Sambo Championship (Cyprus, May 2021), DASS-21 was translated to Russian, Lithuanian, Croatian, and Serbian languages, which are already available at http://www2.psy.unsw.edu.au/dass//translations.htm. These athletes were tested during the break of their athletic duties, whereas recreational ones were tested before and after training sessions. All participants volunteered to participate in the study. Testing lasted for about 10 min, with researchers' supervision. There were none of the data rejected due to partial completion of the scale.

### Instruments

The Depression Anxiety Stress Scales-21 (DASSs-21; Lovibond and Lovibond, 1995b) were used to assess psychological distress in elite sambo and recreational athletes. This 21-item self-report scale contains three subscales (each has seven items) for measuring depression, anxiety, and stress, primarily in non-clinical populations of adolescents and adults (authors suggested using it for individuals older than 14 years). Participants indicated how much each item applied to them over the past week on a four-point Likert scale from 0 (*Did not apply to me at all*) to 3 (*Applied to me very much or most of the time*). A total score is calculated as a measure of psychological distress by summing all items.

DASS is based on dimensional and not on the categorical conception of a researched psychological problem. The differences in depression, anxiety, and stress affect different individuals and differences in the degree of the issue.

Although the initial version has 42 items, research on psychometric properties of the 42-item and 21-item versions of DASS in a clinical and non-clinical population showed that the DASS-21 has the same factor structure, with DASS-21 demanding less time (double) for testing, so it is recommended for assessing aforementioned emotional states in the non-clinical population (Antony et al., 1998). Therefore, when using the DASS-21, the rule is to multiply the obtained scale scores by two so that they can be compared to the DASS normative data and to other published DASS data, which in this case was done.

Due to the low time demand of the scale and the fact that it is easy to fill out and is publicly available, this scale is one of the most used scales when it comes to emotional assessment due to its metric characteristics, which were also confirmed in our case.

### Statistical Analyses

All applied analyses were performed using the software Statistical Package for Social Science (SPSS), version 20 (SPSS, Chicago, IL). Initially, descriptive statistics (means and standard deviations) for the entire sample and also for specific subsamples (male/female, elite sambo/recreational athletes) was conducted. Normality of distribution was checked (skewness, kurtosis, and Kolmogorov–Smirnov test), internal consistency (Cronbach's alpha), intercorrelations for subscales, and total score DASS-21 within the researched sample. Due to distribution not being normal, appropriate nonparametric measures were taken to compare the DASS-21 scale and subscale scores by sports type/gender in the total sample, with significance being set at *p* < 0.05.

## Results

In Table 1 are shown means and standard deviations for the three subscales and the total score of the DASS-21 (multiplied by 2), indicators of distribution normality (skewness, kurtosis, and Kolmogorov–Smirnov test), Cronbach's alpha coefficients, correlations (Pearson's r) among the three subscales, and general distress in the total sample.

**Table 1:** Descriptive statistics and distribution of DASS-21 in the total sample.

	**M**	**SD**	**Sk**	**Ku**	K–S^*^	**α**	**2**	**3**	**4**
1. DASS-21 depression	4.51	6.12	2.386	8.181	0.231	0.780	0.461^*^	0.614^*^	0.788^*^
2. DASS-21 anxiety	5.86	6.38	1.804	4.824	0.179	0.775		0,705^*^	0.839^*^
3. DASS-21 stress	10.96	10.96	0.609	−0.258	0.123	0.820			0,930^*^
4. DASS-21 total	21.33	18.40	1.290	3.346	0.123	0.897			

*M, mean; SD, standard deviation; Sk, skewness; Ku, kurtosis; K–S, value of Kolmogorov–Smirnov test*.

* *Values of Kolmogorov–Smirnov tests are significant (p < 0.01)*.

*α, Cronbach's alpha coefficients; 2, 3, 4 = intercorrelations*.

Values of standard errors for skewness and kurtosis are .156 and .310, respectively, *p < 0.01.

Mean of the total athletes' sample in DASS-21 score was 21.33 (SD = 18.40); between males and females, no statistically significant difference was found: general distress [(M_m_= 20.69; SD_m_ = 16.09 to M_f_= 22.02; SD_f_ = 20.65), depression (Mm = 4, 63; SDf = 6.59; SDm = 5.66 to Mf = 4.37 to Mf = 4.37), anxiety (M_m_= 5.69; SD_m_ = 5.61 to M_f_= 6.05; SD_f_ = 7.12), and stress (M_m_= 10.38; SD_m_ = 7.76 to M_f_= 11.59; SD_f_ = 9.89)]. Females displayed a trend in several above average values when compared to males u in all assessed, except for depression.

Normality tests (skewness, kurtosis, and Kolmogorov–Smirnov test) show that data are not normally distributed (values: from .123 to .231; all *p* < 0.01); a majority of athletes had lower scores in examined subscales of depression, anxiety and stress, and in general distress, with a great deviation from normality, especially in the subscale of depression.

Cronbach's alpha coefficients obtained from the athletes whose values vary from 0.775 (for anxiety subscale) to 0.897 (for general distress) show very good consistency of the scale and are in agreement with the original manual, from 0.73 to 0.81 (Lovibond and Lovibond, 1995a), and other studies that have used this instrument all over the world (Bottesi et al., 2015, from 0.74 to 0.92; Jovanović et al., 2014, from 0.77 to 0.92).

Intercorrelations between specific subscales of DASS-21 in this research range from 0.461 to 0.705; the obtained correlation coefficients are of mild intensity and could be considered as expected if we take into consideration that researched aspects have a common cause in genetic and other factors of vulnerability, which have a tendency to be common for all emotional states and not specific for one. Acquired correlation total score of general distress with depression subscale (r = 0.788), anxiety subscale (r = 0.839), stress subscale (r = 0.930), despite the magnitude of coefficient, also points toward the common cause of all the researched variables of distress and toward differences which further lead to conclusions that some events in the environment that cause bad emotions within an overall population are partly specific. There are several studies where the factorial structure of DASS-21 was explored. It was shown that a trifactorial model with the unknown factor of general distress best describes the structure of the scale (Henry and Crawford, 2005). This model is applied in our research as well.

Table 2 shows descriptive statistics (mean rank, Mann–Whitney U, and p) after comparing depression/anxiety/stress scores as well as psychological distress total score between elite sambo athletes and recreational athletes in the total sample.

**Table 2:** Comparison of depression/anxiety/stress scores as well as psychological distress total score between elite sambo athletes and recreational athletes in the total sample (values of mean rank, Mann–Whitney U test, and *p*).

	**Elite sambo**	**Recreational**	**U**	p^*^
	**(*n* = 105)**	**(*n* = 140)**		
	**Mean rank**			
**Total athletes sample**
DASS-21 depression	110.36	132.48	6,022.50	0.013
DASS-21 anxiety	111.93	131.30	6,188.00	0.032
DASS-21 stress	102.41	138.44	5,188.00	0.000
DASS-21 total	104.44	136.92	5,401.00	0.000

* *p < 0.05*.

All tested differences between elite sambo and recreational athletes were statistically significant; recreational athletes had a higher score on the depression scale, anxiety and stress, and a general distress score than sambo athletes. Average values affirm accuracy gained through Mann–Whitney test comparing sambo and recreational athletes: in depression subscale M_s_= 3.03 (SD_s_ = 3.794) and M_r_= 5.61 (SD_r_ = 7.217), in anxiety subscale M_s_ = 5.22 (SD_s_ = 6.236) and M_r_ = 6.34 (SD_r_ = 6.459), in stress subscale M_s_= 8.67 (SD_s_ = 8.769) and M_r_= 12.69 (SD_r_ = 8.552), and in general distress M_m_ = 16.91 (SD_m_ = 16.11) and M_f_= 24.64 (SD_f_ = 19.35). Although data are not normally distributed, means and standard deviations should be seen as exploratory, but these values are valuable for comparison with other studies that used similar testing tools.

On the level of the total sample, no statistically significant gender differences were found. However, it is possible that these differences were skewed since no data were obtained on the type of sports males and females were engaged in. Nevertheless, these differences might explain differences between sambo athletes and recreational athletes in psychological distress. Table 3 shows differences in depression/anxiety/stress scores as well as psychological distress total score between elite sambo athletes and recreational athletes within the male subsample and within the female subsample (mean rank, Mann–Whitney U, and p).

**Table 3:** Differences in depression/anxiety/stress scores as well as psychological distress total score between elite sambo athletes and recreational athletes within the male and female subsamples (values of *mean rank, Mann–Whitney U test*, and *p* for males and females).

	**Elite sambo**	**Recreational**	**U**	p^*^
	**(*n* = 52)**	**(*n* = 75)**	
	**Mean rank**			
**Male athletes sample**
DASS-21 depression	64.06	63.96	1,947.00	0.988
DASS-21 anxiety	60.91	66.14	1,789.50	0.425
DASS-2 stress	56.70	69.06	1,570.50	0.061
DASS-21 total	58.38	67.89	1,658.00	0.151
	**Elite sambo**	**Recreational**		
	**(*****n*** **=** **53)**	**(*****n*** **=** **65)**		
**Female athletes sample**
DASS-21 depression	47.92	68.95	1,108.50	0.001
DASS-21 anxiety	52.38	65.31	1,345.00	0.038
DASS-21 stress	47.13	69.58	1,067.00	0.000
DASS-21 total	47.01	69.68	1,060.50	0.000

* *p < 0.05*.

Based on the results shown in Table 3, within male subsamples, differences between elite sambo and recreational athletes in self-assessment of psychological distress were not statistically significant, which was similarly found in the total sample. It is noticeable that the trend of higher values is found within recreational athletes in comparison with sambo athletes, except for the subscale of depression. Some inconsistencies were found when conducting a *t*-test, which is sometimes recommended for nonparametric data (Skovlund and Fenstad, 2001), with sambo athletes in the subscale of anxiety having slightly higher values when compared to recreational athletes, with none of the values being statistically significant [in depression subscale M_s_= 4.27 (SD_s_ = 4.534) and M_r_= 4.48 (SD_r_ = 6.334), in anxiety subscale M_s_= 5.81 (SD_s_= 6.630) and M_r_= 5.60 (SD_r_ = 4.832), in stress subscale M_s_= 9.08 (SD_s_ = 8.274) and M_r_= 11.28 (SD_r_ = 7.298), and in general distress M_s_= 19.15 (SD_s_ = 17.03) and M_r_= 21.76 (SD_r_= 15.45)].

However, elite sambo athletes show significantly lower scores on all examined variables—less depression, less anxiety, less prone to stress, and cope with psychological distress better when compared to females who are recreationally active. Comparing average values of obtained results between sambo and recreational athletes, all aspect of unpleasant emotional states were confirmed and for general distress: in depression subscale M_s_= 1.81 (SD_s_ = 2.362) and M_r_= 6.46 (SD_r_ = 8.076), in anxiety subscale M_s_= 4.64 (SD_s_= 5.828) and M_r_= 7.20 (SD_r_ = 7.888), in stress subscale M_s_= 8.26 (SD_s_ = 9.291) and M_r_= 14.31 (SD_r_ = 9.606), and in general distress M_s_= 14.71 (SD_s_ = 14.99) and M_r_= 27.97 (SD_r_ = 22.73).

This paper has several relevant limitations. When four subsamples were compared (male and female sambo, male and female recreational athletes), female elite sambo athletes were less depressed and anxious and less prone to stressogenic reactions and had the lowest scores of psychological distress within the total sample. Unlike them, female recreational athletes had the highest values of all examined variables within the total sample screened. Also, female recreational athletes are much more prone to stress than males (*t* = −2.115; *p* < 0.036) when compared with sambo athletes, and males are significantly less stressed than females (*t* = 3.493; *p* < 0.01). This large gap in the display of psychological distress symptoms depends on whether they are engaging in sambo or are recreationally active to more mean values for the female subset and no gender differences in the total sample. Heterogeneity of the female subset has caused to derive all of the statistically significant differences.

## Discussion

Studies of variations in the prevalence of psychological distress in elite sambo and recreational athletes are rare, and this might be due to the theme itself and due to the specificity of the sports sample, especially elite sambo athletes who are not adequately represented in the literature and virtually nonexistent in the sports psychology. Sambo athletes aim to strengthen their body and mind and to use it against the opponent, which can also reflect on mental health. On the other side, the psychological distress of sambo athletes was compared with recreational athletes who are also aware of physical activity and are active habitually. They definitely feel the benefits of physical activity on their mental health, which makes them different than the overall population. When comparing elite sambo athletes and recreational athletes in psychological distress, we expected that the quality of the elected sport had a significant influence.

Indeed, acquired results confirm this premise; elite sambo athletes scored significantly lower compared to recreational athletes in all examined variables of psychological distress—DASS-21 depression subscale score, DASS-21 anxiety subscale score, DASS-21 stress subscale score, and DASS-21 general distress (total) score differentiated elite sambo and recreational athletes. A possible explanation for these results might lie in the level of engagement in a particular sport. For elite athletes, an elevated level of stress and tension is a sort of normal environment that they are used to, so they can cope better with it, unlike their recreational counterparts. Everyday encounters with high demands, pressures, and expectations have upgraded the level of mental toughness in elite athletes. Therefore, what some non-athletes or recreational athletes perceive as tough or difficult to overcome, professional athletes might perceive the same experience in a milder magnitude. This does not mean that other athletes do not develop a mechanism to cope with pressures that are specific to a particular position that they hold in a given sport or physical activity, but differences in the effectiveness of overcoming and controlling psychological distress between elite athletes and the others exist according to our study. There is no doubt that the differences in psychological distress between athletes and non-athletes could be explained by the presence of positive emotions in athletes that could counter the negative ones. However, the control group within this study was comprised of recreational athletes who gladly engage in physical activity so that those positive emotions might be even more displayed in recreational athletes (Wienke and Jekauc, 2016), which excludes this as a confounding factor. Considering that both subpopulations belong to the athletic population, differences between them in terms of emotional expression are likely caused by the specifics of the sport or physical activity they are engaged in. As a sport, sambo can provide its participants tools to cope with problems whereby their mental health is maintained while dealing with everyday problems. Whether this mental strength stems from the ability to confront an opponent or to confront yourself, or both, might be answered in one of the future studies where the special emphasis would be placed on combat sports and elements of these sports in conjugation with psychological. With that being said, it should be noted that the majority of sambo athletes engage in rapid weight loss due to tactical but also psychological advantages (self-respect, satisfaction, etc.) (Drid et al., 2021; Figlioli et al., 2021). This might be the key reason why elite sambo athletes cope with psychological distress better.

When comparing our results obtained on a total sports sample with the results obtained using the same scale, DASS-21, in the general population (e.g., Jovanović et al., 2014; Bottesi et al., 2015; Sariçam, 2018), we could not notice any regularity in terms of values on particular subscales. However, when separately comparing elite sambo athletes with recreational athletes from our research with the results of other research in which DASS-21 was used, it is noticeable that recreational athletes self-perceive unpleasant emotional states more in line with the general population than elite sambo athletes (Jovanović et al., 2014; Bottesi et al., 2015). Higher average values found in females on the scale of psychological distress compared with male counterparts were not confirmed in this study. Gender studies of mental health suggest a magnified presence of psychological stress in females when compared to males. However, gender differences studies that used DASS offer inconsistent results, often indicating no gender differences (e.g., Norton, 2007; Mahmoud et al., 2010; Apóstolo et al., 2012; Bottesi et al., 2015). In this study, the obtained results can be justified for a sport-participating sample, a factor known to help with mental health issues. This was also shown in our study, where female athletes showed better mental health when compared to the general population and when compared to male athletes—whereby equal results were found. Thus, if there is a difference between males and females in psychological distress in the overall population, it is not noticeable in the athletic population. Therefore, taking part in sports can induce more psychological benefits in females than males, at least in the researched aspects of the study.

In this study, gender dependence of a specific type of sports activity in psychological distress was noted. Within the female sample, female sambo athletes showed lower scores in depression, anxiety and stress subscales, and general distress scale than female recreational ones. In male participants, there are no differences between elite sambo and recreational athletes in psychological distress, just like in the total sample. Notably, females who practice sambo have the lowest score in scales when compared to other subsets (female recreational, males sambo, and recreational), while recreational athletes show the highest score in a subscale of depression, anxiety, stress, and general distress scale. Unlike male samples, which are quite homogenous when it comes to psychological distress (both elite sambo and recreational athletes), in female sample results show that specifics between sports might have a particular effect on mental health.

Our interpretation of the mentioned findings in women refers to the fact that sambo in terms of mental health is most likely an upgrade of something that in other types of physical activity they cannot adopt and refers to the supply of resources to cope with psychological distress. Unlike them, we assume that in male sambo athletes, it is just a continuation of the natural environment which means playing their gender role as a fighter who protects the family from external challenges—he imposes himself as someone who is strong, and who can and resists these challenges. Thus, females through sambo improve their mechanisms of coping with challenges and unpleasant emotional states, while in males, this shift is not significant.

The obtained results should not be generalized on elite athletes' population or athletes' population, since the study was conducted specifically in sambo athletes. Likewise, this holds true for recreational athletes, since the age gap in selected individuals was very narrow. Our control group was a part of the institutionalized and structured group that engaged in habitual physical activity, which implicates that they might be aware of the effect of physical activity on mental health. When interpreting data, we have to notice that the championship was held in the midst of the COVID-19 pandemic and associated precautionary measures (Gentile et al., 2021), which could have influenced the variables.

## Conclusion

Data acquired in this study in terms of the prevalence of psychological distress in athletes suggest that they are vulnerable to a range of mental health issues that may be related to both sport and non-sport factors. Moreover, the results showed gender and specific-sport dependence of examined mental health problems. Females were found to be a vulnerable subsample, especially recreational female athletes, not elite-level ones. Future epidemiological and interventional studies should explore optimal strategies to identify mental health needs based on specific sport activity, especially in gender.

## Data Availability Statement

The raw data supporting the conclusions of this article will be made available by the authors, without undue reservation.

## Ethics Statement

The study obtained ethical approval from the Faculty of Sport and Physical Education, University of Novi Sad, Serbia (Ref. No. 46–06-02/2020–1) and it was conducted according to the Helsinki Declaration. Furthermore, all sambo athletes gave written informed consent with complete information about the study and questioners provided by the investigator. The patients/participants provided their written informed consent to participate in this study.

## Author Contributions

TT, SE, and PD contributed to the conception and design of the study. TT, BŽ, NL, NZ, BG, ER, TBT, and AB organized the database. TT, NZ, BG, and ER performed the statistical analysis. TT, NZ, AB, and PD wrote the first draft of the manuscript. All authors contributed to the manuscript revision and read and approved the submitted version.

## Funding

This work was supported by the Provincial Secretariat for Higher Education and Scientific Research (142-451-2594).

## Conflict of Interest

The authors declare that the research was conducted in the absence of any commercial or financial relationships that could be construed as a potential conflict of interest. The reviewer AG declared a shared affiliation with several of the authors, NL and AB to the handling editor at time of review.

## Publisher's Note

All claims expressed in this article are solely those of the authors and do not necessarily represent those of their affiliated organizations, or those of the publisher, the editors and the reviewers. Any product that may be evaluated in this article, or claim that may be made by its manufacturer, is not guaranteed or endorsed by the publisher.

## References

[B1] Akesdotter C. Kentt,ä G. Eloranta S. Franck J. (2020). The prevalence of mental health problems in elite athletes. J. Sci. Med. Sport. 23, 329–335. 10.1016/j.jsams.2019.10.022 31806359

[B2] Antony M. M. Bieling P. J. Cox B. J. Enns M. W. Swinson R. P. (1998). Psychometric properties of the 42-item and 21-item versions of the depression anxiety stress scales in clinical groups and a community sample. Psychol. Assess. 10, 176–181. 10.1037/1040-3590.10.2.176

[B3] Apóstolo J. L. A. Tanner B. A. Arfken C. L. (2012). Confirmatory factor analysis of the Portuguese depression anxiety stress scales-21. Rev. Lat. Am. Enfermagem. 20, 590–596. 10.1590/S0104-11692012000300022 22991123

[B4] Bauman J. (2016). The stigma of mental health in athletes: are mental toughness and mental health seen as contradictory in elite sport? Br. J. Sports Med. 50, 135–136. 10.1136/bjsports-2015-095570 26626270

[B5] Bottesi G. Ghisi M. Altoè G. Conforti E. Melli G. Sica C. (2015). The Italian version of the Depression Anxiety Stress Scales-21: Factor structure and psychometric properties on community and clinical samples. Compr. Psychiatry. 60, 170–181. 10.1016/j.comppsych.2015.04.005 25933937

[B6] Brody L. R. Hall J. A. (2008). “Gender and emotion in context,” in Handbook of Emotions (3rd ed.), Lewis, M., and Haviland, J. (eds). New York: Guilford. p. 395–408.

[B7] Chang C. J. Putukian M. Aerni G. Diamond A. B. Hong E. S. Ingram Y. M.. (2020). Mental health issues and psychological factors in athletes: detection, management, effect on performance, and prevention: american medical society for sports medicine position statement. Clin. J. Sport Med. 30, e61–e87. 10.1097/JSM.0000000000000817 32000169

[B8] Diener E. Wirtz D. Tov W. Kim-Prieto C. Choi D. Oishi W.. (2010). New well-being measures: short scales to assess flourishing and positive and negative feelings. Soc. Indic. Res. 97, 143–156. 10.1007/s11205-009-9493-y

[B9] Drid P. Figlioli F. Lakicevic N. Gentile A. Stajer V. Raskovic B.. (2021). Patterns of rapid weight loss in elite sambo athletes. BMC Sports Sci. Med. Rehabil. 13, 39. 10.1186/s13102-021-00267-3 33853685 PMC8045259

[B10] Figlioli F. Bianco A. Thomas E. Stajer V. Korovljev D. Trivic T.. (2021). Rapid weight loss habits before a competition in sambo athletes. Nutrients. 13, 1063. 10.3390/nu13041063 33805862 PMC8064378

[B11] Gentile A. Trivic T. Bianco A. Lakicevic N. Figlioli F. Roklicer R.. (2021). Living in the “bubble”: athletes' psychological profile during the sambo world championship. Front. Psychol. 12, 657652. 10.3389/fpsyg.2021.657652 34122241 PMC8187578

[B12] Gouttebarge V. Castaldelli-Maia J. M. Gorczynski P. Hainline B. Hitchcock M. E. Kerkhoffs G. M.. (2019). Occurrence of mental health symptoms and disorders in current and former elite athletes: a systematic review and meta-analysis. Br. J. Sports Med. 53, 700–706. 10.1136/bjsports-2019-100671 31097451 PMC6579497

[B13] Gouttebarge V. Jonkers R. Moen M. Verhagen E. Wylleman P. Kerkhoffs G. (2017). The prevalence and risk indicators of symptoms of common mental disorders among current and former Dutch elite athletes. J. Sports Sci. 35, 2148–2156. 10.1080/02640414.2016.1258485 27894209

[B14] Henry J. D. Crawford J. R. (2005). The short-form version of the Depression Anxiety Stress Scales (DASS−21): Construct validity and normative data in a large non-clinical sample. Br. J. Clin. Psychol. 44, 227–239. 10.1348/014466505X29657 16004657

[B15] Jovanović V. Gavrilov-Jerković V. Žuljević D. Brdarić D. (2014). Psychometric evaluation of the depression anxiety stress scales−21 (DASS−21) in a Serbian student sample. Psihologija. 47, 93–112. 10.2298/PSI1401093J

[B16] Kotnik B. Tušak M. Topič M. D. Leskošek B. (2012). Some psychological traits of Slovenian Olympians (Beijing 2008) – a gender comparison. Kinesiol. Slov. 18, 5–18.

[B17] Lakicevic N. Mani D. Paoli A. Roklicer R. Bianco A. Drid P. (2021). Weight cycling in combat sports: revisiting 25 years of scientific evidence. BMC Sports Sci. Med. Rehabil. 13, 1–6. 10.1186/s13102-021-00381-2 34906212 PMC8670259

[B18] Lakicevic N. Roklicer R. Bianco A. Mani D. Paoli A. Trivic T. (2020). Effects of rapid weight loss on judo athletes: a systematic review. Nutrients. 12, 1220. 10.3390/nu12051220 32357500 PMC7281976

[B19] Leach L. S. Christensen H. Mackinnon A. J. Windsor T. D. Butterworth P. (2008). Gender differences in depression and anxiety across the adult lifespan: the role of psychosocial mediators. Soc. Psychiatry Psychiatr. Epidemiol. 43, 983–998. 10.1007/s00127-008-0388-z 18575787

[B20] Li P. De Bosscher V. Pion J. Weissensteiner J. R. Vertonghen J. (2018). Is international junior success a reliable predictor for international senior success in elite combat sports? Eur. J. Sport Sci. 18, 550–559. 10.1080/17461391.2018.1439104 29490566

[B21] Lovibond S. H. Lovibond P. F. (1995a). Manual for the Depression Anxiety Stress Scales. Sydney: Psychology Foundation. 10.1037/t01004-000

[B22] Lovibond S. H. Lovibond P. F. (1995b). The structure of negative emotional states: comparison of the depression anxiety stress scales (DASS) with the beck depression and anxiety inventories. Behav. Res. Ther. 33, 335–343. 10.1016/0005-7967(94)00075-U 7726811

[B23] Mahmoud J. S. R. Hall L. A. Staten R. (2010). The psychometric properties of the 21-item Depression, Anxiety, and Stress Scale (DASS-21) among a sample of young adults S. Online J. Nurs. Res. 10, 21–34. 10.4314/afrrev.v12i2.1322008979

[B24] Norton P. (2007). Depression Anxiety and Stress Scales (DASS−21): Psychometric analysis across four racial groups. Anxiety Stress Coping. 20, 253–265. 10.1080/10615800701309279 17999228

[B25] Prinz B. Dvorák J. Junge A. (2016). Symptoms and risk factors of depression during and after the football career of elite female players. BMJ Open Sport Exerc. Med. 2, e000124. 10.1136/bmjsem-2016-000124 27900184 PMC5117078

[B26] Purcell R. Gwyther K. Rice S. M. (2019). Mental health in elite athletes: increased awareness requires an early intervention framework to respond to athlete needs. Sports Med.-Open. 5, 1–8. 10.1186/s40798-019-0220-1 31781988 PMC6883009

[B27] Rice S. M. Purcell R. De Silva S. Mawren D. McGorry P. D. Parker A. G. (2016). The mental health of elite athletes: a narrative systematic review. Sports Med. 46, 1333–1353. 10.1007/s40279-016-0492-2 26896951 PMC4996886

[B28] Sariçam H. (2018). The psychometric properties of Turkish version of Depression Anxiety Stress Scale-21 (DASS-21) in health control and clinical samples. J. Cogn. Psychother. 7, 19–30. 10.5455/JCBPR.274847

[B29] Schaal K. Tafflet M. Nassif H. Thibault V. Pichard C. Alcotte M.. (2011). Psychological balance in high level athletes: gender-based differences and sport-specific patterns. PLoS ONE. 6, e19007. 10.1371/journal.pone.0019007 21573222 PMC3087722

[B30] Skovlund E. Fenstad G. U. (2001). Should we always choose a nonparametric test when comparing two apparently nonnormal distributions? J. Clin. Epidemiol. 54, 86–92. 10.1016/S0895-4356(00)00264-X 11165471

[B31] Werner K. Gross J. J. (2004). “Emotion regulation and psychopathology: A conceptual framework,” in, Emotion regulation and psychopathology, Kring, A., and Sloan, D. New York, NY: Guilford Press. p. 13–37.

[B32] Wienke B. Jekauc D. (2016). A qualitative analysis of emotional facilitators in exercise. Front. Psychol. 7, 1296. 10.3389/fpsyg.2016.01296 27621718 PMC5002432

[B33] Wylleman P. Rosier N. De Knop P. (2015). “Transitional challenges and elite athletes' mental health,” in Health and Elite Sport: Is high performance sport a health pursuit, Baker, J., Safari, P., and Fraset, J. T. London: Routledge. p. 99–116.

